# Mesoaortic entrapment of a left inferior vena cava

**DOI:** 10.4103/0971-3026.59758

**Published:** 2010-02

**Authors:** Ashish Gupta, Nitish Naik, Gurpreet Singh Gulati

**Affiliations:** Department of Cardiovascular Radiology, All India Institute of Medical Sciences, Ansari Nagar, New Delhi - 110 029, India; 1 Department of Cardiology, All India Institute of Medical Sciences, Ansari Nagar, New Delhi - 110 029, India

**Keywords:** Inferior vena cava, multidetector computed tomography, ultrasound

## Abstract

A persistent left inferior vena cava (IVC) is a rare anomaly, with a reported incidence of only 0.2-0.5%. When present, it courses between the superior mesenteric artery and the aorta to continue as the right IVC, similar to the course of a left renal vein (LRV). This anomaly is usually asymptomatic, but there may be vague abdominal complaints if the IVC is compressed in the mesoaortic angle. Although symptomatic compression of the LRV (anterior nutcracker syndrome) is well recognized, there has been only one report in the literature of a similar compression of a persistent left IVC. Because of its rarity, this anomaly may be missed or mistaken for other conditions on imaging. An accurate diagnosis is crucial as the presence of this anomaly may have implications for surgical treatment of aortic lesions or placement of an IVC filter. Magnetic resonance angiography and, more recently, multidetector computed tomography scan, can provide an exquisite three-dimensional demonstration of vascular abnormalities.

## Introduction

The left renal vein (LRV) may get compressed as it courses between the superior mesenteric artery (SMA) and the aorta (through the area also known as the mesoaortic angle). This condition, known as the nutcracker syndrome, presents with nonspecific abdominal complaints and is well described in the literature. A left inferior vena cava (IVC) is a rare vascular anomaly. Although it has a course similar to the LRV, a nutcracker-like phenomenon for a left IVC is extremely rare and has only been reported once in the literature.[1] Clinically, it can mimic more common pathologies. It can also interfere with surgical/interventional treatment of aortic or IVC lesions. Imaging plays an important role in the diagnosis of this condition. Although color Doppler USG can help identify the anomalous IVC, complete three-dimensional demonstration, which is necessary for precise surgical treatment, is best accomplished with MRI angiography (MRA) or CT angiography (CTA).

We herein describe the imaging features in a patient with a left IVC that was severely narrowed in the mesoaortic angle.

## Case Report

**Figure 1 (a,b):**
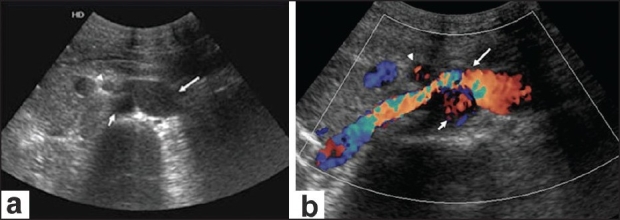
Axial USG image (a) just below the superior mesenteric artery (SMA) (arrowhead) origin shows an oval anechoic structure (long arrow) adjacent to the aorta (short arrow). Color Doppler (b) at the same level shows that the lesion is vascular (long arrow) and is compressed [turbulence is seen as a mixture of colors, higher velocities indicated by higher intensity of color, (orange)] as it courses between the aorta (short arrow) and the SMA (arrowhead)

**Figure 2 (a-c):**
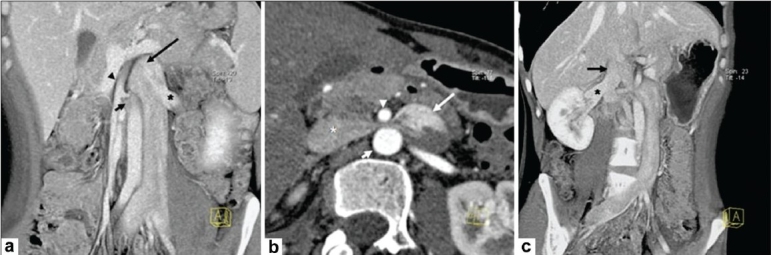
CT angiogram. Coronal oblique maximum intensity projection (MIP) image (a) shows a dilated left inferior vena cava (IVC) (long arrow) receiving the left renal vein (LRV) (*) and entering the mesoaortic angle [between the superior mesenteric artery (SMA) (arrowhead) and the aorta (short arrow)]. The iliac veins and LRV are dilated. Axial thin MIP image (b) shows the dilated left IVC (long arrow) compressed between the aorta (short arrow) and the SMA (arrowhead) as it crosses to the right to become the right IVC (*). Coronal oblique thick MIP image (c) shows the right IVC (long arrow) receiving the right renal vein (*) and coursing toward the heart

A 30-year-old man presented to the local physician with left lumbar pain of 15 days' duration. The pain was gradual in onset and moderate in severity, and there was radiation to the back; there were no obvious aggravating or relieving factors. The routine laboratory investigations were normal. Abdominal USG revealed an anechoic oval lesion adjacent to the aorta and the SMA [Figure 1a]. Subsequently, a Doppler study at our institute showed this to be a dilated left IVC, with turbulent, high-velocity flow as it coursed between the SMA and the aorta [Figure 1b]. For better delineation, CTA was performed on a multidetector dual-source CT scanner (SOMATOM Definition, Siemens, Erlanger, Germany) using standard protocols. The abdominal aorta was normal. The dilated left IVC, after receiving the LRV, dipped between the SMA and the aorta [Figure 2a]. The IVC was significantly narrowed within the mesoaortic angle [Figure 2b], but then received the right renal vein to continue as a normal-sized right IVC [Figure 2c]. The LRV and both iliac veins were dilated. The other intra- abdominal structures were normal.

The patient was advised surgery but did not return for treatment.

## Discussion

Embryogenesis of the IVC is an extremely complex process. It is formed by three paired embryonic veins; namely, the posterior cardinal, the subcardinal and the supracardinal veins in chronologic order of appearance. These veins anastomose and then regress (except for the portions that take part in the formation of the IVC). The mature IVC is a composite structure of these veins and their anastomoses. The hepatic segment of the IVC is formed by vitelline veins and the suprarenal segment is formed by the right subcardinal vein. The anastomosis between the subcardinal and the supracardinal veins develops into the renal segment of the IVC. The right supracardinal vein gives rise to the infrarenal segment.[2] IVC anomalies result from abnormal regression or abnormal persistence of embryonic veins. A persistent left IVC occurs due to the regression of a right-sided supracardinal vein and the persistence of a left-sided supracardinal vein. It has a reported incidence of 0.2-0.5%.[3] The left IVC usually crosses the midline anterior to the aorta in the mesoaortic angle (similar to the normal course of a LRV) and joins the right renal vein to form the right IVC.

The nutcracker syndrome, which was originally described for the compression of an LRV within a narrow mesoaortic angle can therefore be expected to occur with a left IVC as well. This, however, is extremely rare, with only one case reported till date.[1] Catheter angiography was earlier the investigation of choice in such cases but the advent of noninvasive angiography with CT scan and MRI has now made it possible to obtain an accurate, three-dimensional, demonstration of vascular anomalies.[4] With CTA, it is also possible to reconstruct images in any desired plane, which is of great help when planning the appropriate treatment strategy. The imaging criteria for the diagnosis are as follows: (1) the ratio of the anteroposterior distance between the SMA and the aorta to the LRV diameter should be <1:5[5] and (2) renal vein collaterals should be present. The hemodynamic significance of the compression is confirmed on venography when there is a pullback gradient across the narrowing of more than 3 mm Hg.[6]

It is important to be aware of this abnormality as it can lead to abdominal pain, hematuria, ureteral/peripelvic varices, proteinuria or varicocele.[1,7] On imaging, a nutcracker phenomenon with a left IVC can mimic left para-aortic lymphadenopathy, a mass lesion or even aortic aneurysm. A left IVC can also pose significant procedural problems during surgery for aortic aneurysm.[8] For interventionists, it may make infrarenal placement of an IVC filter difficult to perform via a transjugular approach.[8]

Depending on the clinical presentation, the nutcracker syndrome can be managed conservatively, surgically or by an endovascular approach. Surgical approaches include renal vein bypass, renal vein transplantation, SMA transposition, nephropexy and autotransplantation of the kidney.[7,9] Endovascular treatment (balloon angioplasty and stenting) has also been attempted.

To conclude, mesoaortic compression of a left IVC is an extremely rare anomaly and an unusual cause of abdominal complaints. Imaging with CTA helps exclude the other lesions that can mimic this condition and can accurately define the anomalous anatomy for surgical planning.
